# Towards a barrier-free anthropomorphic brain phantom for quantitative magnetic resonance imaging: Design, first construction attempt, and challenges

**DOI:** 10.1371/journal.pone.0285432

**Published:** 2023-07-12

**Authors:** Mikail Kraft, Slavka Ryger, Ben P. Berman, Matthew E. Downs, Kalina V. Jordanova, Megan E. Poorman, Samuel D. Oberdick, Stephen E. Ogier, Stephen E. Russek, Joseph Dagher, Kathryn E. Keenan

**Affiliations:** 1 National Institute of Standards and Technology, Physical Measurement Laboratory, Boulder, Colorado, United States of America; 2 The MITRE Corporation, McLean, Virginia, United States of America; 3 Hyperfine, Inc, Guilford, Connecticut, United States of America; 4 Department of Physics, University of Colorado, Boulder, Colorado, United States of America; Medical University of Vienna: Medizinische Universitat Wien, AUSTRIA

## Abstract

Existing magnetic resonance imaging (MRI) reference objects, or phantoms, are typically constructed from simple liquid or gel solutions in containers with specific geometric configurations to enable multi-year stability. However, there is a need for phantoms that better mimic the human anatomy without barriers between the tissues. Barriers result in regions without MRI signal between the different tissue mimics, which is an artificial image artifact. We created an anatomically representative 3D structure of the brain that mimicked the T1 and T2 relaxation properties of white and gray matter at 3 T. While the goal was to avoid barriers between tissues, the 3D printed barrier between white and gray matter and other flaws in the construction were visible at 3 T. Stability measurements were made using a portable MRI system operating at 64 mT, and T2 relaxation time was stable from 0 to 22 weeks. The phantom T1 relaxation properties did change from 0 to 10 weeks; however, they did not substantially change between 10 weeks and 22 weeks. The anthropomorphic phantom used a dissolvable mold construction method to better mimic anatomy, which worked in small test objects. The construction process, though, had many challenges. We share this work with the hope that the community can build on our experience.

## 1. Introduction

Magnetic resonance imaging (MRI) can be used for the measurement of specific distances and volumes or for assessment of other quantitative properties such as relaxation times or water diffusion. Increasingly, quantitative MRI properties are being tested as tools to assess response to treatment [1], for diagnostics [2], and for treatment planning [3]. MRI reference objects or phantoms can be used for many purposes, such as quantitative assessment of MRI scanners or method performance. Many of these objects are constructed using rigid materials with geometric containers of known sizes that hold stable materials with specific quantitative MRI properties. However, these geometric objects are not representative of human anatomy, and they can create artifacts in the images that are not present for *in vivo* imaging. Therefore, development of anthropomorphic reference objects, or phantoms, that capture anatomic features with greater fidelity is needed.

Anthropomorphic phantoms have been developed to represent human cardiac vessels [4], breasts [5], the pelvis, [6] and brains in partial form [7–13]. Partial brain representations include 2D slice phantoms such as those by Gopalan et al. [7] and Saotome et al. [8], and the polyvinyl alcohol (PVA) brain based on Colin27 [9]. Other partial brain representations contain different cavities in the phantom representative of the different brain structures (e.g., the Martinos Center “Angel” phantom [10]). However, they do not always contain an interior representative of brain structure [11, 12] or are designed for applications other than MRI [13]. For example, Magsood and Hadimani designed a phantom for transcranial magnetic stimulation without a barrier between the brain tissue and surrounding cerebrospinal fluid [14]. They used a polylactic acid (PLA) mold, dissolved with acetone, to create the brain tissue (there was no distinction between white and gray matter). These efforts are summarized by Filippou & Tsoumpas [15], McGarry et al. [16], Valladares et al. [17], and Crasto et al. [18]. Recent work prints the white matter and gray matter structures directly using naturally derived polymers [19]. This is a promising approach; however, it is not yet demonstrated for a complete brain mimic. Creating a 3D anthropomorphic brain structure is a challenging problem, and each group has focused effort on the aspect of the phantom that matters most to their problem or application. No 3D anthropomorphic brain structure suitable for whole-brain quantitative MRI has been developed.

The main goal of this study was to create an anatomically accurate MRI reference object of the brain with an interior representative of brain structures and without barriers between the white matter and gray matter. The phantom was designed for 3 T using materials that will be stable on the order of months or, ideally, a few years. We considered different strategies to create the barrier-free anthropomorphic phantom and use dissolvable molds for the final design, similar to [14]. This work aims to replicate features relevant to quantitative relaxometry and susceptibility mapping, though the method could be extended to other quantitative MRI features. Additionally, while the primary goal is development for 3 T, stability of the phantom was tested at 64 mT on an FDA-approved, portable MRI system that was more accessible for routine scanning than the 3 T system.

## 2. Methods

### 2.1 Construction of the anthropomorphic brain phantom

#### 2.1.1 Concept for the anthropomorphic brain phantom

**Fig 1:**
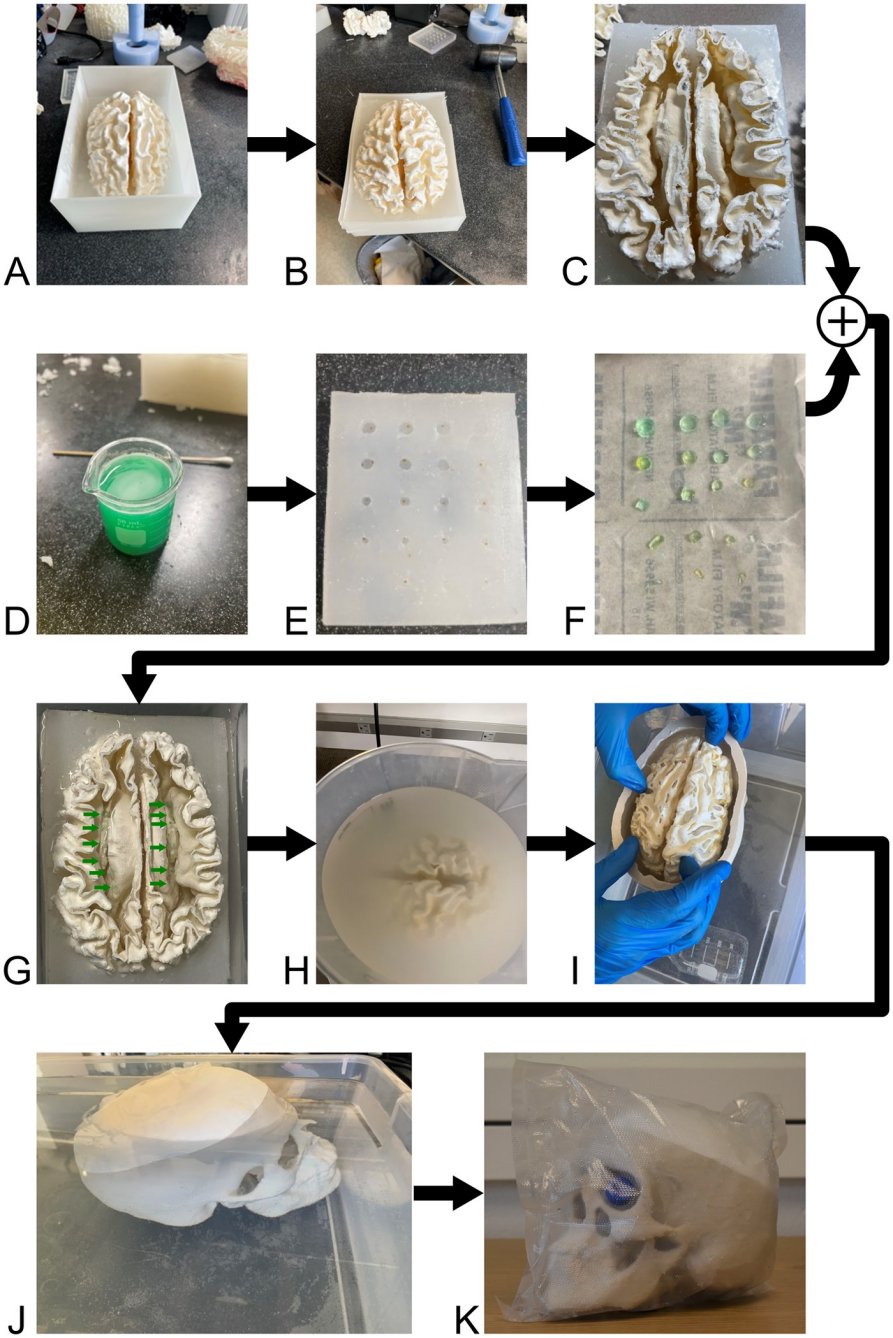
Photographs of the construction process for the anthropomorphic phantom. The white matter shell was 3D printed (A), and then a silicone holder was created to facilitate the agarose gel assembly (B). The top of the white matter shell was removed (C) for filling. Immediately prior to filling with the agarose gel white matter mimic, the nano-iron oxide disc inclusions were created from agarose and iron-oxide nanoparticles. Food coloring was used to make positioning within the white matter easier (D). The nano-iron oxide discs were made in a silicone mold (E). The nano-iron oxide discs were removed from the disc mold (F). The white matter shell was filled with the white matter agarose gel mimic, and the nano-iron oxide discs (marked with green arrows) were carefully positioned (G). Then the white matter gel was allowed to solidify. Next, the 3D printed ABS shell was dissolved using acetone (H). The white matter was positioned within the 3D printed skull (I) and filled with the gray matter agarose gel mimic. Finally, the top of the skull was fixed to the bottom part of the skull, and the remaining space was filled with gray matter agarose gel mimic (J). The complete anthropomorphic phantom, within the vacuum sealed bag, is shown in (K).

To create anatomically accurate MRI reference objects with features for quantitative analysis, the phantom requirements were: (a) to comprise both white matter and gray matter, preferably with a fold-like structure for at least one of white or gray matter; (b) to be encased in a human-sized skull-like object; (c) to include intentional susceptibility features to mimic microbleeds; (d) to be clear of barriers that can create signal voids and susceptibility artifacts in the MRI image; and (e) to include sinus cavities, which present challenges to accurate quantitative susceptibility mapping. To achieve these features, a sacrificial white matter mold with the fold-like structure was 3D printed and filled with doped agarose gel to mimic white matter relaxation properties; nano-iron oxide particles in agarose gel were used for intentional susceptibility features within the white matter; the white matter mold was dissolved leaving only the white matter gel; a skull including sinus cavities was 3D printed; the white matter gel was inserted; and the surrounding space was filled with the gray matter doped agarose gel. Throughout the process, care was taken to avoid air bubbles in the agarose gel preparation and phantom construction. A visual summary of the construction is shown in Fig 1. Complete details of the construction procedure are available in the S1 Appendix.

The A.A. Martinos Center’s “MGH Angel 001” voxel head model was selected as the base for the 3D anthropomorphic model [10]. The MGH Angel 001 model has white matter, gray matter, and skull segments defined, which were used to generate 3D models for the white matter mold and skull/gray matter mold. Details on this process are given in the S1 Appendix.

#### 2.1.2 White matter mimic: Materials and 3D printing

The agarose white matter mimic was designed with a 3 T target T1 of 780 ms and T2 of 65 ms based on measurements by Jiang et al. [20] (though the range of T1 relaxation times reported in the literature for 3 T is approximately 700 ms to 1700 ms [20–24]). To achieve this, we used 1% agarose with 0.510 mmol/L NiCl_2_ and 0.033 mmol/L MnCl_2_ using a similar method to Gopalan et al. [7] (Table 1). We used agarose, rather than agar, which required different compositions of agarose with NiCl_2_ and MnCl_2_ to achieve the T1 and T2 values. It was not possible, in our tests, to modulate T1 and T2 to the desired values using agarose alone. The white matter gel was created using a sacrificial mold, printed in acrylonitrile butadiene styrene (ABS). Additional details follow in sections 2.1.4 and 2.1.5 and in the S1 Appendix.

**Table 1:** Chemical compositions for the tissue mimic materials.

Tissue mimic	Agarose	NiCl_2_ (mmol/L)	MnCl_2_ (mmol/L)	10 nm Fe_3_O_4_ iron-oxide nanoparticles
White matter	1%	0.510	0.033	0
Gray matter	1%	0.558	0.001	0
Nano-iron oxide gel discs	1%	0.50	0	0.2 μL per disc
Product information	Agarose Type I A6013, Sigma-Aldrich, St. Louis, MO, USA	Nickel(II) chloride hexahydrate, Sigma-Aldrich, St. Louis, MO, USA	Manganese(II) Chloride Tetrahydrate, Sigma-Aldrich, St. Louis, MO, USA	Sigma-Aldrich, St. Louis, MO, USA

#### 2.1.3 Microbleed mimics

Nano-iron oxide gel discs were included in the white matter gel to mimic microbleeds. The objective was to use the minimum amount of iron oxide nanoparticles that could be detected using susceptibility imaging methods. An initial phantom had obvious dipole patterns resulting from the nanoparticle concentration (S2.2 Fig in S2 Appendix). Based on that early work on a clinical 3 T system and other tests on a small-bore 3 T system, the concentration of nanoparticles was lowered by an order of magnitude for this implementation (S2.3 Fig in S2 Appendix).

The nano-iron oxide gel discs were created using polydimethylsiloxane (PDMS) molds with dimensions 2 mm thickness and diameters of 2 mm, 3 mm, 4 mm, or 5 mm (Fig 1E) (PDMS product details: SYLGARD 184 Silicone Elastomer Kit, The Dow Chemical Company, Midland, MI, USA). In total 16 discs were created. The starter gel for the inclusions was a 1% agarose gel with 0.5 mmol/L NiCl_2_ to reduce the T1 (Table 1) dyed with green food coloring (Fig 1D). No MnCl_2_ was included because the iron oxide nanoparticles appreciably lower the T2. Superparamagnetic Fe_3_O_4_ nanoparticles with 10 nm average particle size and 5 mg/mL concentration in water were used (Sigma-Aldrich, St. Louis, MO, USA). The nanoparticles are water-soluble and have a surface coating of carboxylic acid. The disc volume was approximately 6.3 μL to 39.3 μL total, each containing 0.2 μL of iron oxide nanoparticles. The iron oxide nanoparticles were mixed into the starter gel and left to set in the PDMS mold. (Agarose will set quickly, in less than one minute for this volume, and the discs could not be left unattended, or significant water evaporation could occur, which would substantially deform the shape and change the solution concentration.) Immediately following solidification, the nano-iron oxide gel discs were removed from the PDMS mold (Fig 1F), sprayed with a silicone-based sealant (Sofsole Heavy-Duty silicone waterproofer (Implus Footcare, LLC Durham, NC, USA)), and inserted into the white matter gel. Based on the work of Kim et al. [25], the silicone-based sealant was used as a barrier to prevent the nano-iron oxide particles from diffusing into the white matter.

#### 2.1.4 White matter mimic: Filling

To avoid the creation of bubbles or striations within the white matter gel, the nano-iron oxide discs were inserted before the white matter gel had time to solidify. The white matter mold was created in two parts. The bottom mold was placed in a silicone holder (Fig 1A–1C, details in S1 Appendix) to stabilize it and enable a single person to fill it with the white matter gel (silicone product details: Ecoflex 00–35 FAST Silicone Rubber Compound, Reynolds Advanced Materials, Denver, CO, USA). The bottom white matter mold was partially filled, then the nano-iron oxide discs were carefully inserted to avoid creating paths or layers that are visible in MR images (Fig 1G). To increase the time available for inserting objects, the white matter mold was placed within a warm water bath, which slowed the cooling of the agarose gel. The nano-iron oxide gels did not visually appear to move or drift within the white matter gel after positioning. After the nano-iron oxide gels were inserted, the white matter mold top was attached to the white matter mold bottom using cyanoacrylate glue (Loctite Gel Control Super Glue, Henkel Corporation, Rocky Hill, CT, USA), and the remainder of the phantom was filled with the white matter mimic through a hole drilled in the top of the white matter mold (details in S1 Appendix). It was important to carefully add the remaining white matter mimic such that the gel already in the mold was not displaced.

#### 2.1.5 White matter mimic: Mold dissolution

After approximately 24 hours, to allow time for solidification of the white matter gel, the ABS white matter mold was dissolved in acetone. The white matter construct was placed on a wire mesh and suspended in a large container of acetone placed on a stir plate (Fig 1H). Approximately every hour, the white matter construct was carefully placed in a water bath and ABS pieces that could be were removed. The acetone bath was refreshed to ensure the solution was not saturated with ABS, and the white matter construct was placed in the refreshed bath. This process continued for approximately four hours to remove as much ABS as possible. Finally, the gel was rinsed with water and set to dry under a fume hood for 30 minutes to remove any excess acetone. A test of the ABS mold and acetone dissolution process is shown in S2.1 Fig in S2 Appendix.

#### 2.1.6 Gray matter mimic: Materials and 3D printing

The gray matter was created in a similar manner to the white matter. Instead of using a sacrificial mold, the gray matter was cast directly inside the interior of the 3D printed skull. In much the same fashion as the white matter mold, the skull/gray matter mold was printed as top and bottom halves to allow easy insertion of the white matter. Because it did not need to be dissolved, the skull was printed in polylactic acid (PLA) (Makerbot PLA for Replicator+, Makorbot Industries, LLC, New York, NY, USA) and was internally sealed using three coats of white Plasti Dip (Plasti Dip International, Blaine, MN, USA) to achieve water tightness.

An agarose gel mimic for gray matter was made using 1% agarose gel, 0.558 mmol/L NiCl_2_, and 0.001 mmol/L MnCl_2_ to achieve the target T1 relaxation time of 1190 ms and T2 of 110 ms, respectively (Table 1). Similarly to the white matter mimic, the grey matter target relaxation times were taken from the measurements of Jiang et al. at 3 T [20]. (The literature range for gray matter is also large, from approximately 1000 ms to 1800 ms [20–24]). The gray matter/skull mold was placed in a warm water bath on a 3D printed stand to stabilize the mold for filling. While being mindful of the volume displacement of the white matter gel, the bottom half of the gray matter mold was filled with some of the prepared gray matter gel. Then, the white matter was placed into the gray matter/skull mold and held in place for five minutes to allow the gray matter gel to partially solidify (Fig 1I). The top half of the gray matter mold was glued onto the mold using cyanoacrylate glue and allowed to dry. The mold was then filled the rest of the way with gel through a hole in the top of the skull piece. Additional warm water was added to the water bath to surround the gray matter-filled skull mold (Fig 1J). The procedure was done in a warm water bath so that the gray matter gel at the bottom of the assembly would integrate with the gray matter gel added after the placement of the white matter gel without a line between the two gray matter gel layers. The entire assembly was left in the warm water bath to slowly cool and set for at least 12 hours to avoid cracking and bubbles.

#### 2.1.7 Final construction

The skull ensemble was left to solidify overnight. Then it was removed from the water bath, allowed to drain for several hours, dried, and coated in silicone (DAP All-Purpose 100% Silicone Adhesive Sealant, DAP Global Inc., Baltimore, MD, USA) at a thickness of approximately 3 mm. The skull ensemble was placed on a stand to allow the silicone to cure for the required 24 hours. The silicone provided the benefit of additional sealing (to prevent water evaporation from the gel) and to act as a subcutaneous fat mimic. At 3 T, the silicone material has a similar T1 relaxation time and chemical shift as fat tissue [26]. A 15 mL tube of deionized water was placed through the sinus cavity to provide a cerebrospinal fluid (CSF) material reference. As a final precaution against dehydration, the entire anthropomorphic head phantom was placed in a bag and vacuum sealed (Fig 1K). The phantom remained in this vacuum sealed bag for 31 weeks until deconstruction.

### 2.2 MRI measurements

#### 2.2.1 MRI measurement at 3 T to assess tissue mimic relaxation properties

MRI measurements were made at 3 T (Siemens Prisma Fit, syngo MR E11 software, Erlangen, Germany) one day after completing the construction process. Detailed sequence parameters for all sequences are in Table 2. Structural images were acquired using 3D MPRAGE and a 1 mm isotropic 3D spoiled gradient echo sequence volume scan. Susceptibility weighted imaging was acquired using a product 3D sequence. Additionally, 3D single-echo and multi-echo gradient echo sequences were acquired, the multi-echo sequence with 6 echoes. The gradient echo phase images were unwrapped using ROMEO [27]. T1 was measured using an inversion recovery method on a 2D spin echo sequence with 10 inversion times. T2 was measured using a single echo 2D spin echo method with 6 echo times. The T1 and T2 measurements were completed at six slices through the anthropomorphic brain phantom.

**Table 2:** 3 T imaging sequences (TE = echo time; TR = repetition time; BW = bandwidth).

Sequence	Resolution	Acquisition Time (mm:ss)	Parameters
3D MPRAGE	0.8 mm isotropic	6:59	TE = 2.07 ms
			TR = 2400 ms
			BW = 240 Hz/pixel
3D spoiled gradient echo	1 mm isotropic	5:09	TE = 1.9 ms
			TR = 6.3 ms
			BW = 650 Hz/pixel
3D SWI	0.86 mm x 0.86 mm x 1.5 mm	6:38	TE = 20 ms
			TR = 27 ms
			BW = 120 Hz/pixel
3D single-echo gradient echo	0.86 mm x 0.86 mm x 1.5 mm	6:20	TE = 16 ms
			TR = 25 ms
			BW = 80 Hz/ pixel
3D multi-echo gradient echo	0.86 mm x 0.86 mm x 1.5 mm	6:20	TE = 2.7 ms, 6.4 ms, 10 ms, 14 ms, 18 ms, 21 ms
			TR = 25 ms
			BW = 500 Hz/pixel
2D spin-echo inversion-recovery	0.98 mm x 0.98 mm x 3 mm	4:02 per TI	TR = 6390 ms
			TI = 35 ms, 75 ms, 100 ms, 125 ms, 150 ms, 250 ms, 1000 ms, 1500 ms, 2000 ms, 3000 ms
2D spin-echo single echo	1.04 mm x 1.04 mm x 3 mm	11:55 per TE	TE = 35 ms, 75 ms, 100 ms, 125 ms, 150 ms, 250 ms, 1000 ms, 1500 ms, 2000 ms, 3000 ms
			TR = 5000 ms

T1 was calculated for each voxel using the lmfit package in Python for the IR model [28]:

$$S_{i}=S_{0}\left|1-\left(1+d\right)e^{-\;\frac{{TI}_{i}}{\;T_{1}}}+e^{-\;\frac{TR}{T_{1}}}\right|$$ (1)

with T1 the target value to fit, inversion time TI, repetition time TR, scale factor for imperfect inversion d, the nominal signal intensity for a voxel S_0_, and measured signal intensity S_i_. T2 map was calculated for each voxel using the lmfit package in Python for the model:

$$S_{i}=S_{0}e^{-\;\frac{TE}{T_{2}}}$$ (2)

with T2 the target value for the fit and echo time, TE. T1 and T2 maps were calculated for the six acquired slices. Then, square regions of interest (ROIs) approximately 3 mm x 3 mm were selected for each of the target tissues (white matter, gray matter and CSF) over the six slices. The mean and standard deviation over the six ROIs are reported.

#### 2.2.2 MRI measurements at 64 mT for stability assessment

Due to ease of access, longitudinal measurements were made at 64 mT on four occasions: immediately following construction and again at 10-, 22-, and 31-weeks post-construction. The 64 mT measurements used a Hyperfine Swoop 1.8 system with research software version rc8.3.1 (for initial and 10-week imaging) and research software version rc8.5.0 (for 22- and 31-week imaging) (Hyperfine, Inc., Guilford, CT, USA). No deep learning was used for the image reconstruction. Detailed sequence parameters for all sequences are in Table 3. The anthropomorphic phantom was stored in the refrigerator between imaging sessions and given 24 hours to come to scan room temperature prior to imaging. T1 was measured using a whole-brain 3D fast spin echo (FSE) inversion recovery experiment with 15 inversion times. T2 was measured using a 3D FSE sequence with 10 echo times. Additionally, we examined the phantom using clinical sequences for brain imaging available on the Hyperfine including FLAIR, T1-weighted and T2-weighted sequences.

**Table 3:** 64 mT imaging sequences.

Sequence	Resolution	Acquisition Time (mm:ss)	Parameters
3D fast spin echo inversion recovery	1.6 mm x 1.6 mm x 5 mm	11:14 per TI	TR = 3000 ms
			TI = 100 ms, 200 ms, 300 ms, 400 ms, 500 ms, 600 ms, 700 ms, 800 ms, 900 ms, 1100 ms, 1300 ms, 1500 ms, 1800 ms, 2100 ms, 2500 ms
3D fast spin echo	1.5 mm x 1.5 mm x 5 mm	17:08	TE = 37 ms, 111 ms, 185 ms, 259 ms, 333 ms, 407 ms, 480 ms, 554 ms, 628 ms, 702 ms
			TR = 3000 ms
FLAIR (clinical)	1.6 mm x 1.6 mm x 5 mm	9:30	TE = 194.4 ms
			TR = 4000 ms
			TI = 1400 ms
T1 Gray/White (clinical)	1.6 mm x 1.6 mm x 5 mm	6:17	TE = 5.96 ms
			TR = 1500 ms
			TI = 300 ms
T2 (clinical)	1.5 mm x 1.5 mm x 5 mm	6:30	TE = 203.6
			TR = 2000 ms

T1 was calculated for each voxel in the same manner as at 3 T (Eq 1). A T2 map was calculated using a Hyperfine mapping protocol that uses SciPy optimize curve_fit in Python for the same model as at 3 T (Eq 2). T1 and T2 maps were calculated for the entire image volume. Then, square regions of interest (ROIs) approximately 3 mm x 3 mm were selected for each of the target tissues (white matter, gray matter and CSF) over six slices through the volume. The mean and standard deviation over the six ROIs are reported.

### 2.3 Deconstruction of the anthropomorphic brain phantom

Following the 31-week imaging, the phantom was deconstructed to examine causes of artifacts observed in the MR images and look for the nano-iron oxide discs. It was removed from the vacuum sealed bag, and it was cut along the central sagittal plane from top of skull to the cervical spine. Additionally, two samples were taken to look for the presence of acetone in the agarose gel mimics: one from a location near the edge of the white matter mimic and one from the gray matter mimic. First, 2 mm– 3 mm of agarose gel was removed from the cut surface, then a biopsy punch was used to remove the sample of white or gray matter mimic, which was placed in a sample holder for nuclear magnetic resonance (NMR) spectroscopy. The samples were each placed in an NMR at 3 T and approximately 20 °C to examine the chemical spectra, specifically for acetone and water. Additional details are in the S1 Appendix.

## 3. Results

Using the procedure described in the Methods, we assembled an anthropomorphic brain phantom and characterized it using MRI at 3 T. Additionally, we made stability measurements at 64 mT on four occasions.

### 3.1 MRI measurement at 3 T to assess tissue mimic relaxation properties

**Fig 2:**
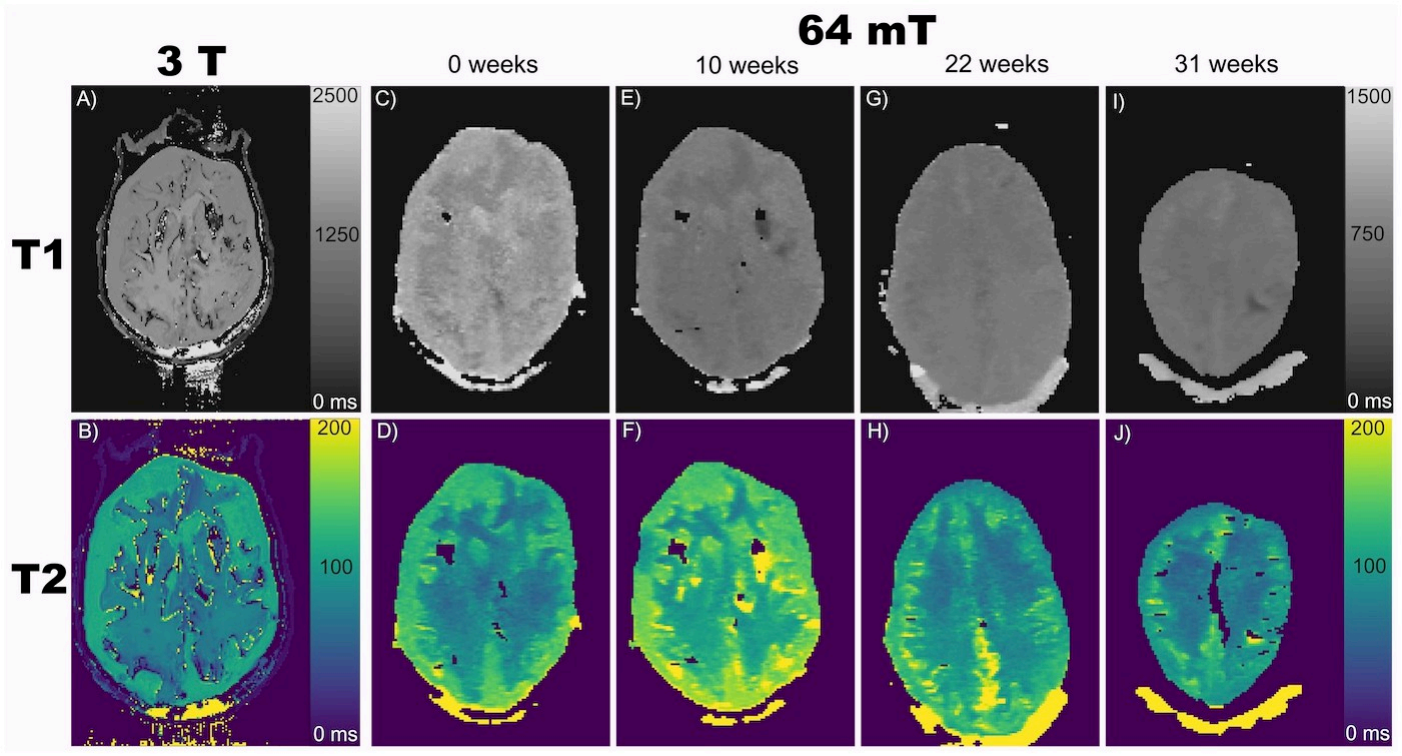
Representative axial T1 and T2 maps acquired at 3 T (A, B) and 64 mT at construction (C, D); 10 weeks (E, F); 22 weeks (G, H); and 31 weeks (I, J) post-construction.

The measured mean T1 and T2 relaxation times for white matter at 3 T, 19.8 °C +/- 0.5 °C, were 1494 ms (181 ms) and 90 ms (10 ms), respectively (the standard deviation over six slices is given in parentheses). For gray matter, the measured mean T1 and T2 relaxation times were 1472 ms (34 ms) and 118 ms (5 ms) at 3 T. The white and gray matter relaxation times are within the range of values reported in the literature for in vivo measurements at 37 °C (Table 4) [20–24]. Representative T1 and T2 maps from 3 T are shown in Fig 2A and 2B.

**Table 4:** Measured relaxation times for white matter, gray matter and CSF mimics at 3 T, 19.8 °C +/- 0.5 °C. The experimental measurements are reported as the mean value over six image slices with the standard deviation in parentheses.

Tissue	T1 (ms)	T2 (ms)	T1 (ms) literature	T2 (ms) literature	Literature reference
White matter	1494 (181)	90 (10)	700–1700	65–75	[20–24]
Gray matter	1472 (34)	118 (5)	1000–1800	80–100	[20–24]
CSF	2840 (212)	1155 (687)	3800–6800		

### 3.2 MRI measurements at 64 mT for stability assessment

While the phantom was only designed to be used at 3 T, the T1 and T2 relaxation times were also measured at 64 mT, 21.5 °C +/- 0.5 °C (Table 5) [29–33] to assess stability. The T1 and T2 relaxation time measurements were repeated at 10 weeks following the original measurement and again 22 and 31 weeks after the original measurement. At 10 weeks, white matter T1 decreased 30%, and T2 was within a standard deviation of the original measurement. Gray matter T1 decreased 22%, and again, T2 was within a standard deviation of the original measurement. At 22 weeks, compared to the week 0 measurements, white matter T1 decreased 24%, and T2 was, again, within a standard deviation of the original measurement. Gray matter T1 decreased 23%, and T2 was within a standard deviation of the original measurement. At 31 weeks, compared to the week 0 measurements, white matter T1 decreased 24%, and T2 was within a standard deviation of the original measurement (both measurements were consistent with the measurements at 22 weeks). Gray matter T1 decreased 27%, and T2 decreased 11%; T2 was no longer within a standard deviation of the original measurement. Representative T1 and T2 maps from 64 mT at the original measurement, 10 weeks, 22 weeks, and 31 weeks are shown in Fig 2C–2J.

**Table 5:** Measured relaxation times for white matter, gray matter and CSF mimics at 64 mT, 21.5 °C +/- 0.5 °C at construction and again at 10, 22, and 31 weeks after construction. The experimental measurements are reported as the mean value over six image slices with the standard deviation in parentheses.

	Original measurement		10-week measurement		22-week measurement		31-week measurement		Literature values		Literature reference
Tissue	T1 (ms)	T2 (ms)	T1 (ms)	T2 (ms)	T1 (ms)	T2 (ms)	T1 (ms)	T2 (ms)	T1 (ms)	T2 (ms)	
White matter	929 (104)	84 (14)	644 (77)	99 (15)	710 (15	85 (12)	704 (10)	88 (3)	270–295	100	[29–33]
Gray matter	1063 (29)	135 (6)	828 (51)	145 (11)	815 (40)	129 (6)	772 (7)	120 (4)	378–400	100	[29–33]
CSF	2698 (600)	1513 (300)	1948 (409)	1534 (302)	1642 (720)	1285 (588)	1957 (85)	1440 (62)			

### 3.3 Microbleed mimics

**Fig 3:**
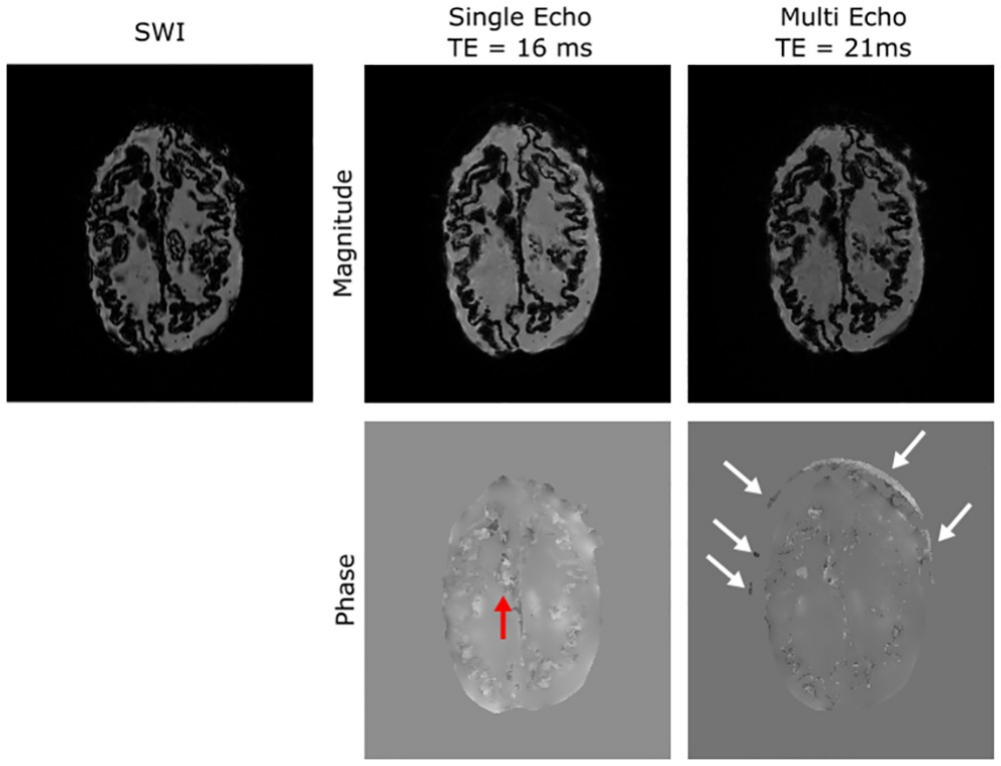
Detection of microbleed mimics. From left to right, a susceptibility weighted image, a single gradient echo acquired (TE = 16 ms), and the final echo from a multi-echo gradient echo (TE = 21 ms). The top row is magnitude images. The bottom row is unwrapped phase images. Phase images were automatically masked in the ROMEO pipeline. In the multi-echo phase image, the masking did not remove all of the 3D printed skull (white arrows). While there are many artifacts in the images, none of these correspond to the location of the nano-iron oxide discs. For example, the red arrow indicates ABS remaining from incomplete removal of the white matter shell.

Performance of the nano-iron oxide discs, which were designed to produce local changes in susceptibility, was assessed using susceptibility weighted imaging (SWI) and 3D single- and multi-echo gradient echo images (Fig 3). These images contained many feature structures, none of which could be obviously correlated with the placement of the nano-iron oxide discs. The SWI and gradient-echo images have a large number of artifacts; however, none of these have the clear dipole structure anticipated from the nano-iron oxide discs.

### 3.4 Assessment of the anthropomorphic phantom construction

**Fig 4:**
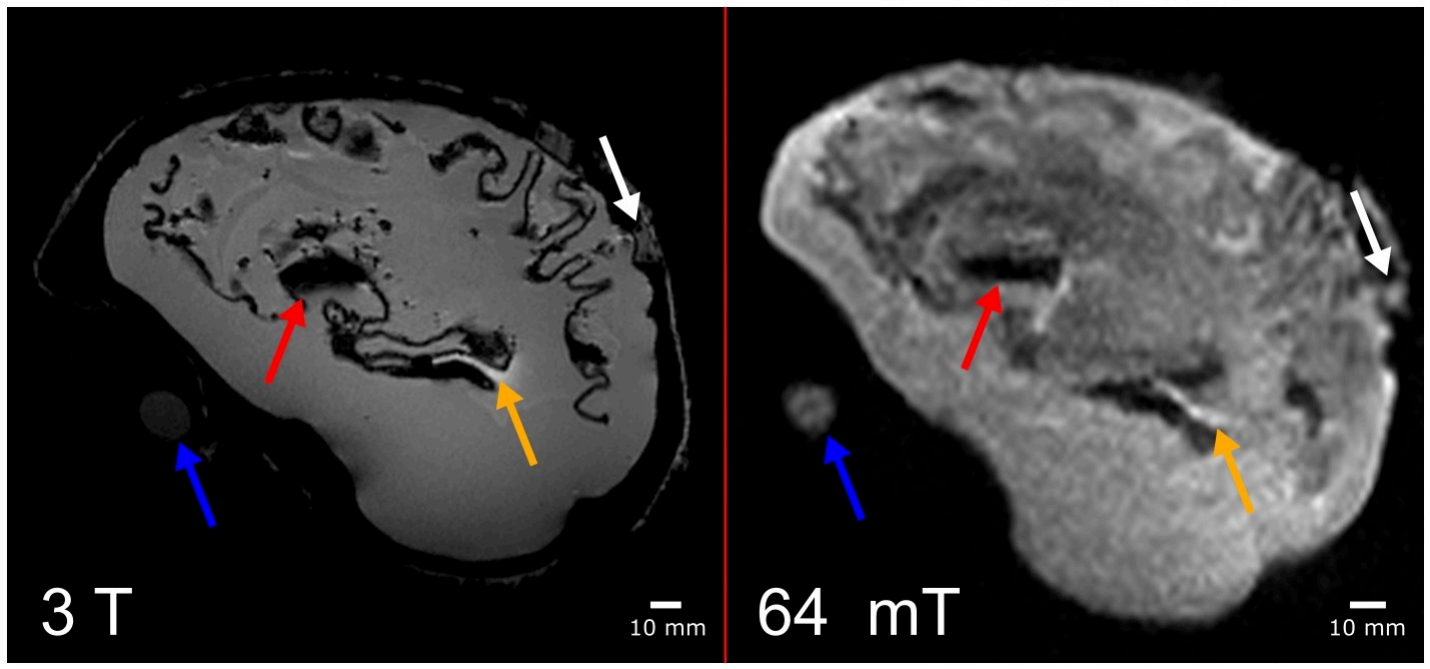
Sagittal MR images of the completed phantom at 3 T (MPRAGE) and 64 mT (FLAIR). In both images the blue arrow shows the water tube; the red arrow points to incomplete removal of the 3D printed white matter shell; the yellow arrow points to remaining silicone from the white matter holder; and the white arrow shows water contained in the 3D printed skull.

**Fig 5:**
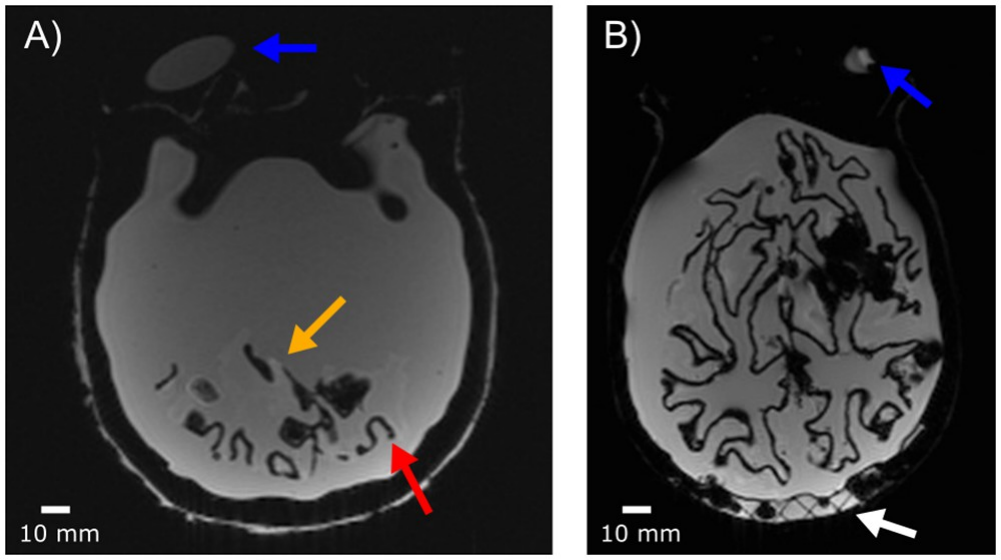
Additional details of the construction flaws. (A) and (B) show the anomalies in more detail on a 1 mm isotropic volume scan (spoiled gradient echo) acquired at 3 T. (A) the red arrow points to leftover 3D printed ABS (dark in the image) and the yellow arrow highlights silicone mold material (bright in the image), and the blue arrows indicate the water tube. (B) water from the assembly process remains in the 3D printed skull shell (white arrow).

An MPRAGE image from 3 T and a FLAIR image at 64 mT are shown in Fig 4, and images from the isotropic volume scan at 3 T are shown in Fig 5. In all images, there are locations of signal void (e.g., at the red arrows in Figs 4 and 5). This is most likely due to incomplete removal of the ABS 3D print structure surrounding the white matter. There were no signal voids nor dipole patterns that could be correlated to the placement of the nano-iron oxide discs. Additionally, there is a hyperintensity within the brain (yellow arrows in Figs 4 and 5), which is most likely silicone material remaining from the base used to stabilize the white matter mold for filling. There is no cerebellum in the anthropomorphic phantom, because it separated from the rest of the white matter prior to final construction of the phantom. Also of note, there is water contained in the 3D-printed skull (indicated by the white arrows in Figs 4 and 5), which was likely trapped within the skull when sealed. By visual comparison of the maps in Fig 2C–2J, it appears that there was some evaporation or redistribution of the water in the 3D printed skull over the 31 weeks.

### 3.5 Deconstruction of the phantom

**Fig 6:**
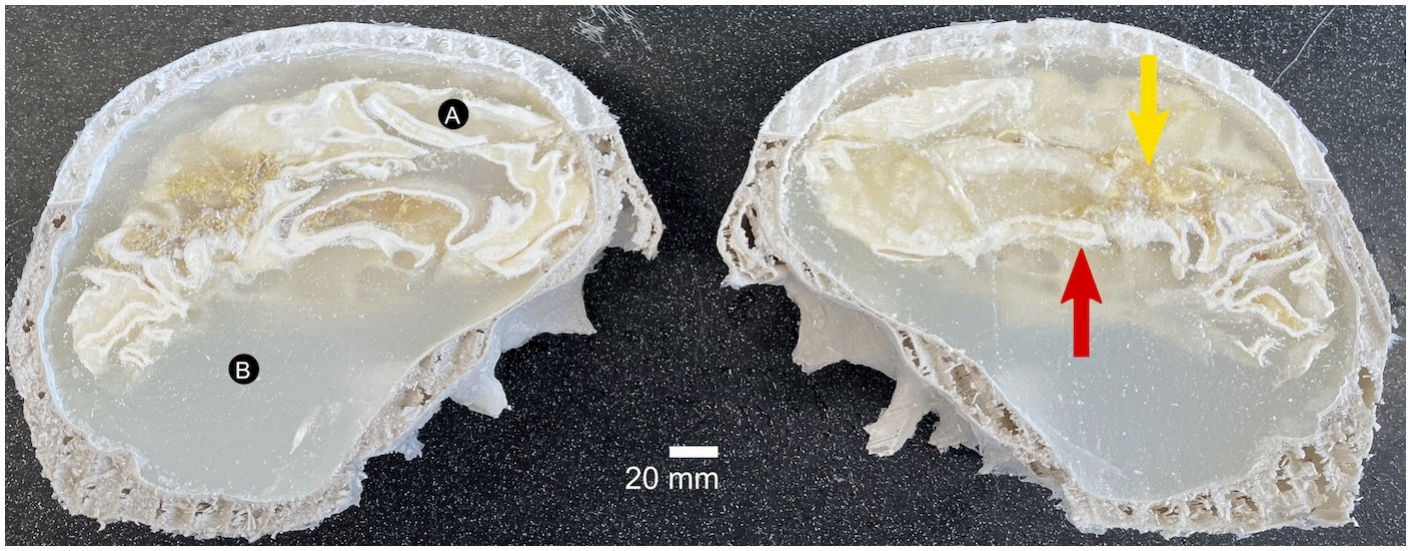
Photo of the anthropomorphic brain cross-section following deconstruction at 31 weeks. A red arrow points to leftover 3D printed ABS, and the yellow arrow highlights silicone mold material. Additionally, the location of the samples for NMR assessment are shown with the black circles for white matter edge (A) and gray matter (B).

A photo of the anthropomorphic brain cross-section is shown in Fig 6. In this figure, the amount of remaining ABS (red arrow) and the remaining silicone mold material (yellow arrow) are apparent. While it cannot be assessed from this photo, there were no visually apparent bubbles in the white or gray matter gels. The green dye used for the nano-iron oxide discs was not visible at the time of deconstruction. Samples were taken below the surface of the gel from two locations: white matter edge (black circle A) and gray matter (black circle B). The NMR spectra for each of these samples had a single peak associated with water (S3.1 Fig in S3 Appendix).

## 4. Discussion

The anthropomorphic phantom presented here achieved some of the intended goals. At 3 T, the T1 and T2 relaxation times for brain tissues were within the range of reported values in the literature; however, the white matter and gray matter had the same T1 relaxation times. Based on measurements at 64 mT, the phantom relaxation properties did not substantially change between 10 weeks and 22 weeks. At 3 T the image resolution and SNR revealed the 3D printed barrier between white and gray matter and flaws in the construction, which were confirmed at deconstruction. Additionally, the susceptibility weighted imaging (SWI) had too many possible features, most likely from remaining ABS and possibly air bubbles in the gel or at gel interfaces with the remaining ABS. The resulting anthropomorphic phantom was moderately successful, and the process can be improved.

Creation of anthropomorphic phantoms for medical imaging is an area of active research, and several groups have tried different approaches and with different goals [7–13, 19, 34]. Gopalan et. al. did extensive work on the tunability of agar using NiCl_2_ and MnCl_2_ to match white and gray matter [7]. We used agarose rather than agar, and we were unable to match the tunability (details in section 4.1.1 and the S2 Appendix). Similar to our effort, Altermatt et. al. worked to create a barrier-free anthropomorphic phantom without mixing between tissue types, though they could not find an MR-invisible solution [34]. Finally, the best solution may be to directly print the tissue mimic materials, as is being pursued by Kilian et. al. [19].

The method used here could be further developed to enable testing of other MRI techniques, rather than microbleed mimics for susceptibility imaging. It should be possible to add features of different, known sizes (e.g., a brain bleed, a tumor, or a microcalcification) to test the accuracy of volume measurement. Additionally, the phantom could be designed to have properties for both MRI and focused ultrasound applications [35]. Finally, future work would incorporate the appropriate permittivity and conductivity properties in the tissue mimic design. We hope that the community can build on this experience.

### 4.1 MRI measurement at 3 T to assess tissue mimic relaxation properties

The materials used in this phantom at room temperature (approximately 20 °C) could replicate reported white and gray matter relaxation properties from in vivo measurements (approximately 37 °C). At 3 T, the T1 relaxation time for gray matter was approximately 24% higher than the targeted value and in the middle of the range of values reported in the literature. The T2 relaxation times for both white and gray matter were approximately 20% higher than the targeted values. T1 relaxation time for white matter was within the range of values reported in the literature, though it was almost twice the targeted value. There was no observed contrast between white and gray matter using MPRAGE, because the T1 relaxation times of the white and gray matter were very similar.

To observe MPRAGE contrast, the gray matter T1 must be greater than the white matter T1. While small (50 mL) test batches of gel yielded the desired relaxation time differences between white matter and gray matter, the final construction did not. Possible reasons include miscalculation of the small volumes used in the test batch, resulting in incorrect scaling up for the larger volume. For example, the MnCl_2_ concentration in the gray matter may be too small to be accurately measured. While test batches were made, the relaxation properties of a larger volume of gel were not measured prior to construction. These are limitations that can be addressed in future work.

### 4.2 MRI measurements at 64 mT for stability assessment

Our goal was to create a phantom with stable relaxation properties for one year. In our phantom, the T2 measurement of white and gray matter was stable over the course of 22 weeks. While measured white matter T1 decreased after the original measurement, the 22-week measurement was within a standard deviation of the 10-week measurement. Similarly, following an initial decrease in measured T1 value, the gray matter was stable from 10 to 22 weeks. By 31 weeks only the white matter T2 measurement was comparable to the original measurement. The changes in T1 may be a result of water moving from the gel to the exterior of the phantom, in which case improvements should be made to prevent water diffusion. However, following an initial settling period, the relaxation properties did stabilize for 11 weeks.

Previously, Altermatt et al. tried three different construction methods to create gray and white matter regions without diffusion between the layers, and they were unable to find a non-MRI visible method [34]. Kim et al. used an aerosol-spray material to create a protective barrier around an agarose gel construct that was then included in a surrounding agarose gel [25]. This barrier did prevent diffusion across the agarose gels over the course of their study; however, we were unable to procure the product in the United States. We used a waterproofing spray between the agarose gel constructs to prevent diffusion, and Plasti Dip to improve the watertightness of the 3D print. Given the lack of stability over the first 10 weeks, it is likely that the waterproofing spray was insufficient to prevent interaction between the white matter and gray matter gels.

While the goal of this phantom was to replicate relaxation properties at 3 T, we can compare the T1 and T2 values to literature reports at 64 mT. In agreement with previous observations that T2 has minimal variation with field strength, the T2 relaxation times for white and gray matter were comparable to the values reported in the literature for approximately 64 mT [29–33]. The phantom materials could not match the T1 relaxation properties of tissue across field strengths. At 64 mT, the T1 relaxation times for white and gray matter were approximately three times greater than the values reported in the literature [29–33]. The agarose gel mimic used here is not suitable for 64 mT. If this method is used to create a phantom at field strengths other than 3 T, different compositions must be used to mimic white matter and gray matter. The creation of singular materials that represent tissue relaxation properties across field strengths is challenging and outside the scope of this project.

### 4.3 White matter mold dissolution challenges

Several steps in the process did not go as planned, or, when scaled up, did not perform in the same manner as in the test situation. It was challenging to dissolve all of the ABS material required for the white matter mold. The test prints for developing the process were completed in house, while the final prints were done by an external company due to the complexity of the print. The external prints used more ABS than the test prints, and it was hard to dissolve the thicker layer of ABS material. The dissolution was stopped before the ABS was entirely removed due to concern of acetone uptake by the white matter gel [36] and to prevent potential safety issues resulting from an acetone-gel. Based on the NMR tests, the acetone did not replace water in the white matter gel. If acetone was present, we would observe both an acetone and water peak with clear separation between the two [37]; however, we only observed a single water peak (S3.1 Fig in S3 Appendix). The cost of the external prints made it prohibitive for us to extensively test the process prior to the final construction. Additionally, there was more variability across these external prints than expected. A test was completed on one external print; however, this test print was more watertight than the second external print, which was used for final construction.

### 4.4 Microbleed mimic challenges

In the final construction, we included nano-iron oxide gel disc features to mimic microbleeds in the brain. Microbleeds are challenging to detect, and thus we tried to use the minimum nano-iron oxide concentration that would be detectable by MRI. In preliminary tests on a 3 T PET-MRI system, the dipole artifacts from the nano-iron oxide discs were apparent (S2.2 Fig in S2 Appendix). The concentration used for this experiment was lower than those preliminary tests and was decided upon after iterations in a simple cylindrical volume of gel and imaging on a pre-clinical 3 T system (S2.3 Fig in S2 Appendix). While the size of the discs was sufficiently large to be detectable at this resolution (disc diameters ranging from 2 mm to 5 mm; image resolution 1 mm), it is possible that the concentration of particles was too low. When the anthropomorphic phantom was deconstructed at 31 weeks, there were not any visually apparent bubbles in the gel. We did observe the presence of the remaining 3D print material and silicone material (Fig 6, red and yellow arrows respectively). We cannot rule out the presence of microbubbles at the interfaces between the gel and the remaining 3D print material. It is possible that the artifacts from the remaining 3D print material occluded the artifacts from the nano-iron oxide discs. Finally, the white matter gel aged longer than the gray matter gel (details in section 4.4 below), which may have led to diffusion of the iron-oxide nanoparticles prior to measurements at 3 T.

### 4.5 Construction challenges

Finally, there were unforeseen challenges in the actual construction. A step was added to the process during the final construction, to let the gray matter-filled-skull cool to room temperature within a water bath. During this step, water entered the 3D printed skull, and the water did not all drain from the skull prior to sealing the phantom. An MRI exam prior to sealing would have allowed observation of the water in the 3D printed skull. Alternatively, coating the exterior of the skull in Plasti Dip prior to placing it in the water bath could prevent water uptake in the skull. Additionally, the final construction process did not occur in a single week-long attempt, as intended, due to a variety of challenges. Unfortunately, this resulted in the separation of the cerebellum from the white matter gel and might point to stability issues with large, uncontained agarose objects. During this time period, the white matter gel was stored in a vacuum sealed bag.

Other approaches to constructing this phantom were also considered. PVA is commonly used in 3D printing as a dissolvable support material. Printing the white matter mold in PVA instead of ABS would allow the mold to be dissolved in water instead of acetone, which has the potential to be easier and produce less waste solvent. This method was not pursued because of concerns that water could absorb into the agarose gel or leach NiCl_2_ and MnCl_2_ from the white matter material, which would lead to geometric distortion and a deviation from the target relaxation times. NiCl_2_ and MnCl_2_ are not soluble in acetone, so leaching was not a concern. 3D printing a soft material (FormLabs Castable Resin for the Form2, Formlabs, Somerville, MA, USA) for the white matter mold was explored. However, the intricate structure of the white matter made it very difficult to peel away the soft mold without also tearing away portions of the white matter gel.

Additional questions arise from the use of 3D printed materials with gels. For example, is the 3D printed material itself stable, or does it interact with the agarose gel over time? It is challenging to prevent evaporation through the 3D printed material, and we tried to address that by sealing the skull first with Plasti Dip, then with silicone and finally by vacuum sealing the entire object. This may have been sufficient. While the T1 relaxation time measured at 64 mT decreased from the original measurement to 10 weeks, which could be due to water evaporation, the T1 did stabilize between 10 weeks and 22 weeks, indicating that water evaporation may not have continued, or was possibly not the reason for the initial change in relaxation time. With advancements in 3D printing technology, it may be possible to create a shell that is more impermeable to water. That material should be tested to ensure it does not interact with the phantom material over time.

In general, fabrication of a hydrated phantom with anatomical features was especially challenging because of the compatibility of various materials with water needed to be considered at every step. These considerations become increasingly complex for multi-component phantoms since many steps are needed to produce different parts of the phantom. For instance, acetone-based dissolution of ABS was chosen to prevent metal salts from leaching into the hydrated agarose and changing the relaxation properties. Also, Plasti Dip was used at various points to inhibit evaporation of water from the final, hydrated phantom for long term stability. While the final phantom had flaws, the process described here produced a hydrated phantom with degrees of multi-component stability. At the very least, this fabrication strategy represents a collection of techniques for manipulating 3D printed materials and additives for engineering a complex, hydrated structure. These can be further optimized for other phantom applications in the future.

## 5. Conclusion

Creation of a faithful anthropomorphic phantom is a process that requires many steps, each corresponding to creation of a certain type of tissue mimic. Therefore, construction of structures that simultaneously capture the intricacy of human anatomic structure and the quantitative relaxation properties of tissue is a challenging process. The approach used here, including dissolvable or sacrificial 3D printed molds, may be more successful when attempting a 2D section phantom rather than a 3D structure. For anyone considering this process or a similar process, patience is one of the keys to success.

## Supporting information

S1 Appendix Phantom construction procedure. This file contains details on the phantom construction process.
(DOCX)

S2 Appendix Preliminary construction tests. Here we summarize different methods that were explored in the construction of the phantom including white matter construction methods (section S2.1), inclusion of nano-iron oxide discs (section S2.2) and tunability of the white and gray matter gels (section S2.3). This appendix includes S2.1, S2.2, S2.3 and S2.4 Figs.
(DOCX)

S3 Appendix NMR assessment for acetone and water in the tissue mimic. Details of the NMR measurements and procedure are included here. This appendix includes S3.1 Fig.
(DOCX)

S4 Appendix Gel preparation details. A protocol for the creation of agarose gels.
(DOCX)
