# Supplementary material for: Towards a barrier-free anthropomorphic brain phantom for quantitative magnetic resonance imaging: Design, first construction attempt, and challenges
Source: PLoS One. 2023 Jul 12;18(7):e0285432. doi: 10.1371/journal.pone.0285432 (PMC10337967; doi:10.1371/journal.pone.0285432)
Supplement: S1 Appendix — This file contains details on the phantom construction process. (DOCX) [file pone.0285432.s001.docx]

S1 APPENDIX: Phantom construction procedure

1. Creation of models for 3D printing

The A.A. Martinos Center’s “MGH Angel 001” voxel head model (https://phantoms.martinos.org/MGH_Angel_001_voxels (10)) was the base for the 3D anthropomorphic model. This model is given as a 3-dimensional matrix within a MATLAB.m file (MathWorks, Natick, MA, USA). Each element in the matrix is valued 1-6, where each value corresponds to a different tissue or feature. For example, if an element is value 1, then that space is air, whereas if an element is value 2, then that space is white matter. We created new matrices isolating each value, thus creating 3D logical arrays for each tissue type. Then, using a MATLAB script from the MATLAB file exchange (https://www.mathworks.com/matlabcentral/fileexchange/27733-converting-a-3d-logical-array-into-an-stl-surface-mesh), the logical array was converted to an .stl file. This .stl creates a solid model from the data. In this case, the .stl for the isolated white and gray matter data were needed. With this solid model, we then created the white matter, gray matter and skull components.

To create a model that can be filled with agarose, a 3D model manipulation tool like Blender (Blender Foundation, Amsterdam, Netherlands) was necessary. An .stl stores 3D data as a mesh of triangles adhering to manifold geometry. To make the model hollow, a thickness was to be applied to the model. The mesh was duplicated, and the duplicated model scaled up or down based on user specification. This created a confined space between the meshes, which was set to the model thickness. With a sufficiently thick model, the center can be hollow (otherwise extensive support structures would be necessary). We used 1-2 millimeters of thickness, depending on the complexity of the structure, and exported as .stl.

To create the 3D printing file, slicer software was used. The slicer software gives the 3D printer instructions for how to print a model and often has other tools, such as adding structural supports to areas susceptible to print failure. It also converts the .stl into a format the 3D printer can understand, giving instructions layer by layer. Many printers use G-code for this. The software and printer we used are *Makerbot Print* and the *Makerbot Replicator+* (Makerbot Industries, LLC, New York, NY, USA), respectively. We note that every slicer software is different, so consult the respective literature on how to apply the print settings.

Below are the specifications we used for printing in ABS:

- Print at 210-250 °C
- If applicable, set the heated bed temperature to 110 °C
- Set 0 % infill
- Set no internal supports
- Set print speed to 30-90 millimeters per second
- Set print dimensions as desired

Upon completion, we carefully removed the 3D printed mold from the printer and cleaned the print per the manufacturer recommended process. (When two fill ports were not included in the design, we drilled two holes approximately 5 mm in diameter for filling.) We tested the printed mold by filling it with water. When water leaked through the mold, the mold thickness was increased, and the print speed set lower. The thickness and print speed above worked for our printer; however, there is variability across 3D printing systems: be sure to note the protocol used to achieve a leak-free model. Note: while the Martinos Center recommends using Plasti Dip to achieve watertightness, we did not use Plasti Dip for the sacrificial molds, because we did not want to complicate the dissolution of the mold.

2. White matter and microbleed construction

Next, the sacrificial white matter mold was filled with the white matter mimic agarose gel. Based on the work of Gopalan et al (7), we tested a combination of agarose, NiCl_2_ and MnCl_2_ combinations to determine the white matter and gray matter mimics. Here, we used agarose rather than agar, because it is less friendly to bacterial growth, even though it is a more expensive material. To achieve the target T1 and T2 relaxation times, we used 1 % agarose with 0.510 mmol/L NiCl_2_ and 0.033 mmol/L MnCl_2_. (Details of the gel preparation process are available in the detailed protocol in the S4 Appendix.) The white matter mimic was poured into the white matter mold and left to set.

Our primary objective was to create a reference object for quantitative relaxometry and susceptibility mapping. To achieve that goal, we included nano-iron oxide gels in the white matter gel to mimic microbleeds (details on the gels are provided in the main text). Objects were quickly inserted into the white matter gel (e.g., the nano-iron oxide discs) to prevent the formation of bubbles or striations that would be visible in the MR images. The white matter mold was placed within a warm water bath, which slowed the cooling of the agarose gel and increased the time available for inserting objects. Then the white matter mold top was attached to the white matter mold bottom using cyanoacrylate glue, and the remainder of the phantom filled with the white matter mimic. It was necessary to carefully add the remaining white matter mimic so as not to displace the gel already in the mold.

3. White matter mold dissolution

To remove the sacrificial ABS mold, it was placed on a wire mesh and suspended in a large container of acetone (Figure 1H). To encourage the ABS to accumulate away from the gel, we used a stir rod and plate set to medium. The process took several hours. Approximately every hour, 50 % of the acetone was replaced to ensure the solution was not saturated with ABS. Once the ABS was entirely dissolved, the gel was carefully removed from the container. Some ABS did linger on the surface of the gel, which was peeled off as much as possible. Then, the gel was set to dry under a fume hood for 30 minutes to remove any excess acetone. We were concerned that leaving the gel exposed any longer would increase the risk of the gel dehydrating.

4. Gray matter mimic

To create the gray matter, similar steps to creating the white matter were followed. However, the gray matter mold was not sacrificial and was not dissolved. Rather, the mold for the gray matter was the skull. Additionally, the white matter was inserted into the gray matter/skull mold. This resulted in two design choices: the gray matter/skull mold was printed as top and bottom halves (to allow easy insertion of the white matter), and it was not printed in ABS. Instead, the gray matter/skull mold was printed using PLA, and as recommended by the Martinos Center, the print was internally sealed using multiple coats of Plasti Dip, touching up areas that seemed like they might need additional coverage. The Plasti Dip was used within a fume hood, and we waited a minimum of 4 hours before proceeding with assembly.

An agarose gel mimic for gray matter was made using 1 % agarose gel, 0.558 mmol/L NiCl_2_, and 0.001 mmol/L MnCl_2_ to achieve the target T1 and T2 relaxation times. The gray matter mold/skull was placed in a warm water bath. The bottom half of the gray matter mold was partially filled with some of the prepared gray matter gel. Then, the white matter was gently placed into the gray matter/skull mold and held in place (Figure 1I). At this point, it was a balance between allowing the gray matter gel to solidify around the white matter and preventing additional bubbles or layering in the gel. After five minutes, the top half of the gray matter mold was glued onto the bottom of the gray matter mold using cyanoacrylate glue and allowed to dry. The mold was then filled completely with gray matter gel. Additional warm water was added to the water bath to surround as much of the gray matter/skull mold as possible (Figure 1J). This enabled the assembly to cool slowly over 12 hours and avoid cracks in the gel.

5. Final construction

The skull ensemble was then removed from the water bath, allowed to drain for several hours, dried off, and coated in a silicone (DAP All-Purpose 100% Silicone Adhesive Sealant, DAP Global Inc., Baltimore, MD, USA) at approximately 3 mm thickness. The skull ensemble was placed on a stand to allow the silicone to cure for the required 24 hours. The silicone coating provided additional sealing (to prevent water evaporation from the gel). A 15 mL tube of deionized water was placed through the sinus cavity to provide a CSF-type material reference. As a final precaution against dehydration, the entire anthropomorphic head phantom was placed in a bag and vacuum sealed (Figure 1K).
