# Supplementary material for: Towards a barrier-free anthropomorphic brain phantom for quantitative magnetic resonance imaging: Design, first construction attempt, and challenges
Source: PLoS One. 2023 Jul 12;18(7):e0285432. doi: 10.1371/journal.pone.0285432 (PMC10337967; doi:10.1371/journal.pone.0285432)
Supplement: S2 Appendix — Here we summarize different methods that were explored in the construction of the phantom including white matter construction methods (section S2.1), inclusion of nano-iron oxide discs (section S2.2) and tunability of the white and gray matter gels (section S2.3). This appendix includes S2.1, S2.2, S2.3 and S2.4 Figs. (DOCX) [file pone.0285432.s002.docx]

S2 APPENDIX – Preliminary construction tests

This document contains three sections on preliminary tests of the phantom construction: S2.1 construction of the white matter, S2.2 Inclusion of the nano-iron oxide discs, and S2.3 White and gray matter gel tunability.

S2.1 Construction of the white matter

To create the white matter, we used a dissolvable, sacrificial mold, which allowed the use of complex geometries that cannot otherwise be used in traditional molding techniques. In traditional molding, the gel would need to be removed from the rigid mold. In all our tests using spherical features approximating brain folds, the gel broke during removal from the mold. We also tried a flexible mold (FormLabs Castable Resin for the Form2, Formlabs, Somerville, MA, USA) that could be peeled away from the gel. In that case, the gel was not stiff and broke when peeling off the flexible mold.

For the dissolvable approach, the mold was 3D printed in acrylonitrile butadiene styrene (ABS). ABS was used because it dissolves if submerged in acetone, an inexpensive and relatively safe solvent. A successful test of this process is shown in Figure S2.1. There is concern that acetone would replace the water in the agarose gel, and we used NMR to look for the presence of acetone in the gel (see details in S3 Appendix).


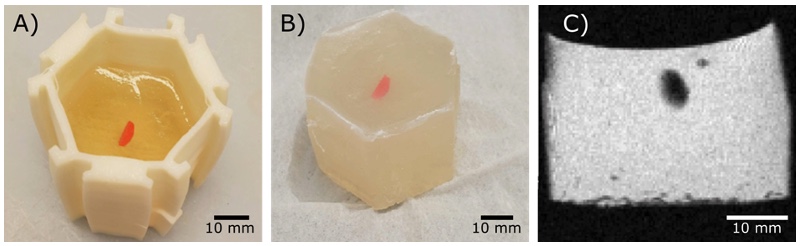


**Figure S2.1: A test object to demonstrate the sacrificial mold.** (A) 3D printed container made from ABS and filled with agarose gel. A pink plastic bead was included as a surrogate for the nano-iron oxide inclusions. (B) The remaining agarose gel after the 3D printed container was dissolved in acetone. (C) MR image of the agarose gel after the ABS container was dissolved. The bottom of the gel shows some ABS fragments that were not sufficiently cleaned prior to pouring in the agarose gel. The small black dot above the bead is an air bubble in the agarose gel.

S2.2 Inclusion of nano-iron oxide discs

We did additional tests to determine a method to place the nano-iron oxide discs within the gel without artifacts. In one approach we made layers of gel around the discs, rather than inserting the discs into an existing gel, and the result was visible layers. Examples from this technique are shown in both a plastic bottle and a 3D printed skull mimic (Figure S2.2). In these preliminary objects there was no attempt to create separate white and gray matter. We also tried placing the discs in the gel and then putting the entire gel into the oven to remove any MR visible evidence of the disc placement; however, this method was not successful (Figure S2.3).


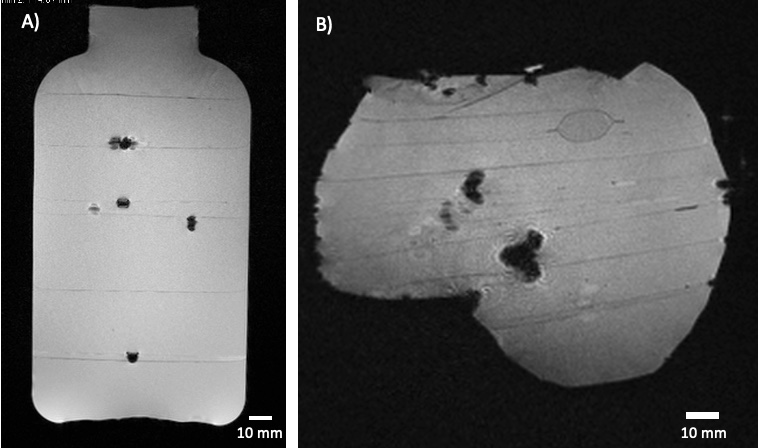


**Figure S2.2: Prototype objects for this project using agarose gel with nano-iron oxide discs.** (A) The first prototype was made in a 1 L Nalgene bottle, and (B) the second prototype was made in a 3D printed container approximating a skull. These are susceptibility weighted images acquired at 3 T on a PET-MRI system. The lines in the agarose gel are obvious and are a result of layering the gel to include nano-iron oxide discs at specific locations and orientations.


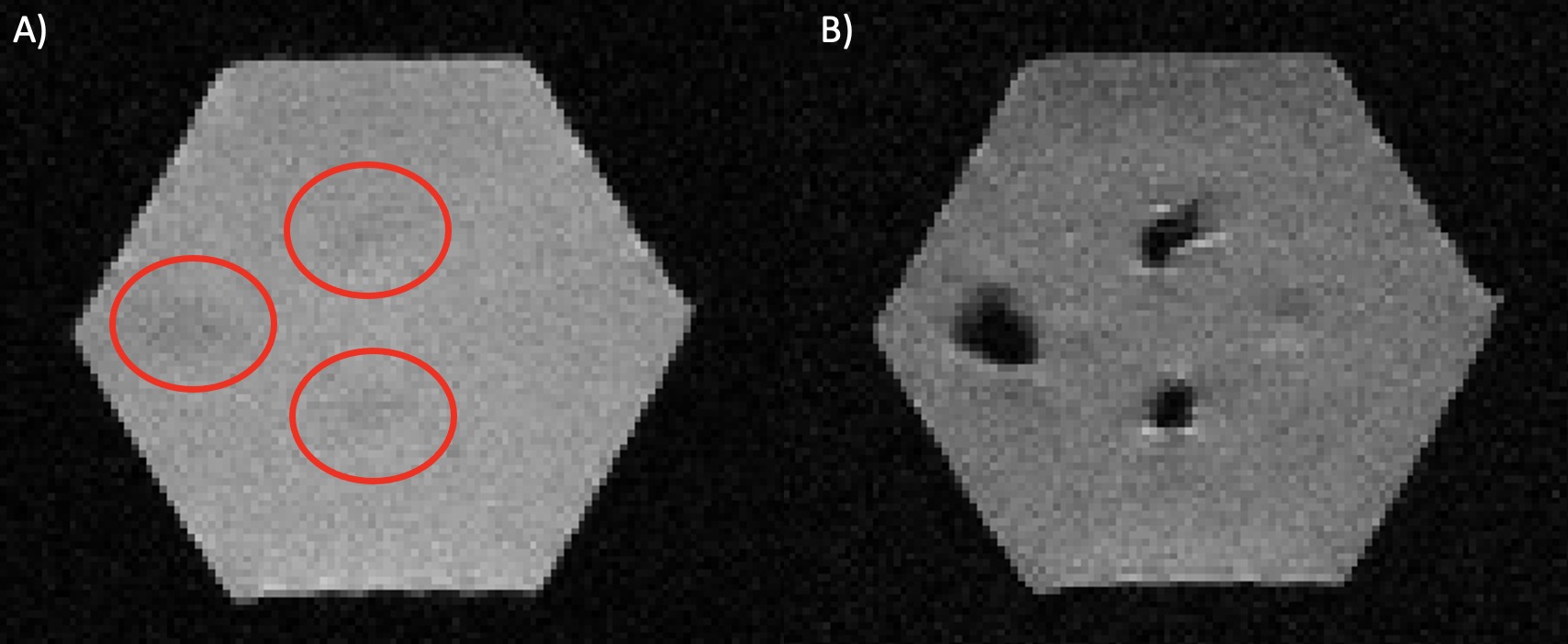


**Figure S2.3: Test of nano-iron oxide disc placement.** One method we considered for placement of the nano-iron oxide discs placed the gel in the oven after positioning the discs. Here we have gradient echo images of slices through the volume of a hexagonal prism. (A) is a slice through the center of the volume containing the discs (red circles). (B) is a slice near the top of the object, where the black artifacts are air pockets at the surface of the object. The use of the oven following placement of the discs did not remove the MR visible effect of placing the nano-iron oxide discs, as seen in (B).

As a result of these preliminary tests, we placed the nano-iron oxide features directly into the large volume of white matter gel, and the white matter gel was placed in a warm water bath. This kept the white matter gel above the solidification temperature and allowed time to carefully place the nano-iron oxide discs.

In these preliminary tests with the nano-iron oxide discs (Figure S2.2), there are black dipole artifacts from the nano-iron oxide discs. For the final construction of the anthropomorphic phantom, the concentration of iron-oxide nanoparticles was reduced to make the nano-iron oxide discs more challenging to detect using susceptibility imaging (similar to Figure S2.3).

S2.3 White and gray matter gel tunability

White and gray matter gel tunability was tested in 50 mL batches with measurements on a small-bore 3 T Agilent system. T1 and T2 relaxation times were measured using the same protocols and analysis detailed in section 2.2.1. These measurements were made at approximately 16.5 °C.

To mimic the white and gray matter relaxation properties, we followed the method of Gopalan et al to identify the appropriate concentrations of paramagnetic salt in gel. We decided to use agarose, rather than agar, because agarose contains fewer substances that support bacterial growth. In our experience, when clean manufacturing processes are followed, agarose gel in a sealed container is resistant to bacterial growth for multiple years. Agar and agarose gels do not have the same relaxation properties, and it was necessary to complete multiple experiments to determine the appropriate concentrations of agarose and paramagnetic salts.

Several experiments were completed using agarose gel concentrations of 1.2 % and 1 % along with various NiCl_2_ and MnCl_2_ concentrations. The resulting T1 and T2 relaxation time measurements for the experiments are shown in Figure S2.4. As expected, the increase in paramagnetic salt concentration decreased the measured T1 and T2 relaxation time values. Repeated gel batches at the same concentrations were not tested, and, along with the small size of the test batches, is a possible source of error in our experiments.


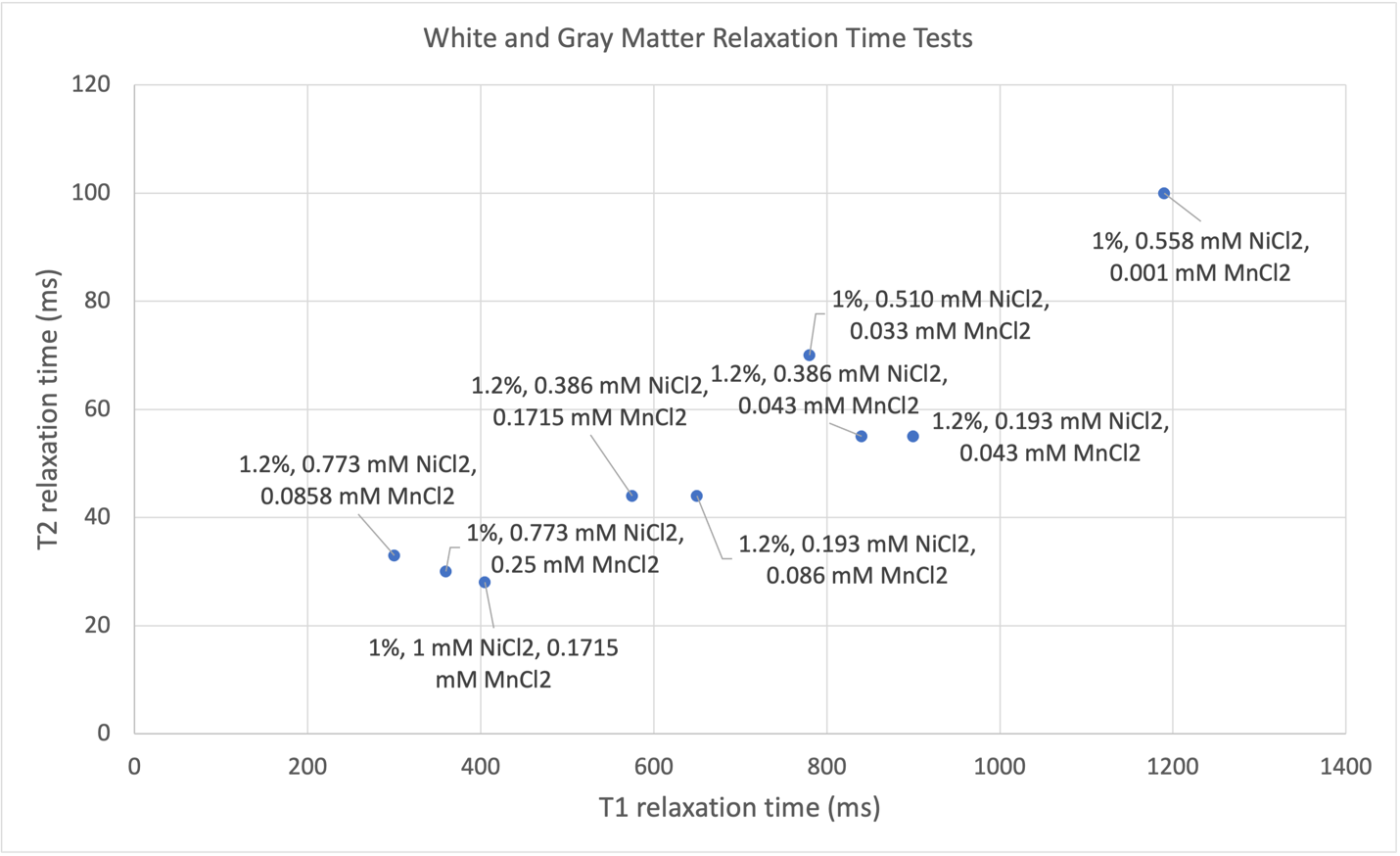


**Figure S2.4: White and Gray Matter Relaxation Time Tests.** Two concentrations of agarose gel (1 % and 1.2 %) with various concentrations of NiCl_2_ and MnCl_2_ were tested to determine the appropriate concentrations for the white and gray matter relaxation times. As expected, the increase in paramagnetic salt concentration decreased the measured relaxation times.
