# Supplementary material for: Towards a barrier-free anthropomorphic brain phantom for quantitative magnetic resonance imaging: Design, first construction attempt, and challenges
Source: PLoS One. 2023 Jul 12;18(7):e0285432. doi: 10.1371/journal.pone.0285432 (PMC10337967; doi:10.1371/journal.pone.0285432)
Supplement: S3 Appendix — Details of the NMR measurements and procedure are included here. This appendix includes S3.1 Fig. (DOCX) [file pone.0285432.s003.docx]

S3 APPENDIX – NMR assessment for acetone and water in the tissue mimic

NMR measurements were made to examine the final construction for the presence of acetone remaining from the dissolution of the white matter mold. The NMR spectra were taken at 3 T at 20 °C on an NMR system dedicated to MRI standards development and MRI calibration. The NMR system, protocols, and analysis are documented in (1, 2). An Oxford 300-44 NMR magnet (Oxford Industries, Oxford, United Kingdom) operating at 3 T (128 MHz) was used with a Doty air-cooled, 3-axis gradient NMR probe tuned to 3 T (Doty Scientific, Columbia, South Carolina, USA). RF power was supplied via a Tomco BT00100-Gamma RF amplifier (Tomco Technologies, Stepney, Australia). The system was controlled with a Tecmag Redstone console (Tecmag, Houston, Texas, USA). For all experiments 1H was measured.

The NMR measurements on the gel samples were obtained using a custom 3 mm NMR cell that mates to a 3 mm biopsy needle and preserves the gel structure. The two samples from the anthropomorphic phantom contained agarose and paramagnetic salts as detailed in the methods (Table 1). The reference sample contained a mixture of 50 % reagent grade acetone and 50 % deionized water sealed in a 2 mm capillary. The data are referenced to the water peak, which is appropriate for MRI studies. The data is archived as part of the NIST MRI Biomarker Measurement Service (3), and the raw data and analysis software are publicly available on request.

In the NMR tests conducted at 31 weeks post-construction of the anthropomorphic phantom, the two gel samples had only a water peak (Figure S3.1).


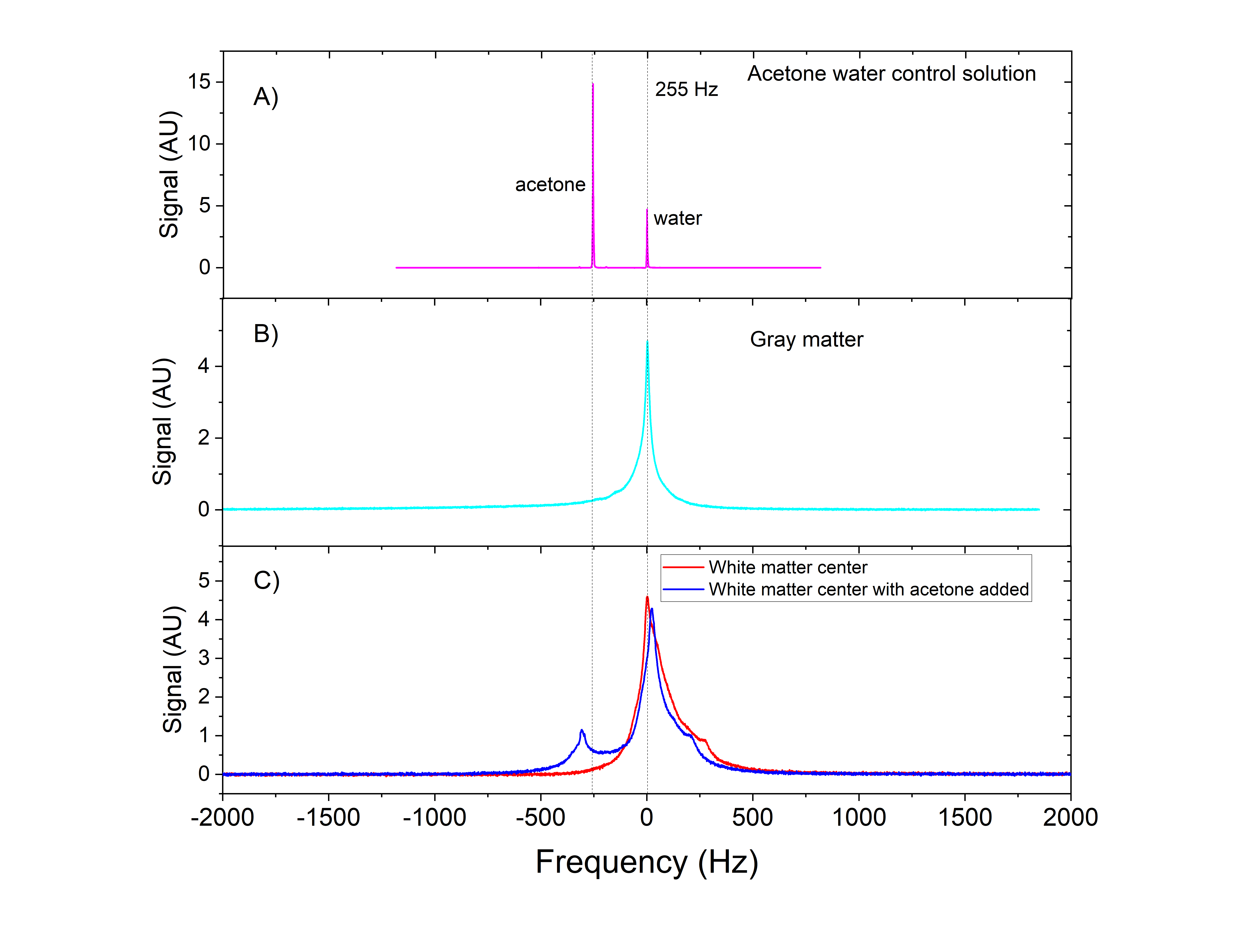


**Figure S3.1: NMR spectra** of (A) a sample containing 50 % acetone and 50 % water with a 255 Hz splitting between the two peaks, (B) a gray matter sample, and (C) a white matter sample with and without acetone added to the sample. The anthropomorphic brain samples contain only a water peak.

References

1. Boss MA, Dienstfrey AM, Gimbutas Z, Keenan KE, Splett JD, Stupic KF, Russek SE. Magnetic Resonance Imaging Biomarker Calibration Service: Proton Spin Relaxation Times 2018; Special Publication (NIST SP) - 250-97. doi: <https://doi.org/10.6028/NIST.SP.250-97>.
2. Boss MA, Keenan KE, Stupic KF, Rentz NS, Stoffer CM, Koepke A, Coakley KJ, Russek SE (2023) Magnetic Resonance Imaging Biomarker Calibration Service: NMR Measurement of Isotropic Water Diffusion Coefficient. (National Institute of Standards and Technology, Boulder, CO), Special Publication (SP) NIST SP 250-100. <https://doi.org/10.6028/NIST.SP.250-100>.
3. <https://www.nist.gov/programs-projects/magnetic-resonance-imaging-mri-biomarker-measurement-service>
