# Supplementary material for: Towards a barrier-free anthropomorphic brain phantom for quantitative magnetic resonance imaging: Design, first construction attempt, and challenges
Source: PLoS One. 2023 Jul 12;18(7):e0285432. doi: 10.1371/journal.pone.0285432 (PMC10337967; doi:10.1371/journal.pone.0285432)
Supplement: S4 Appendix — A protocol for the creation of agarose gels. (DOCX) [file pone.0285432.s004.docx]

S4 APPENDIX - Gel Preparation Details

1. Use a beaker or flask that is 2 - 4x the target volume.
2. Weigh out the agarose and add it to the flask. Add the appropriate amount of deionized water.
3. To hydrate, swirl the beaker and suspend the agarose in solution. Alternatively, you can use a stir bar and stirring plate to rapidly mix the solution but remember to remove the stir bar before microwaving.
4. Let the agarose hydrate a minute or two before proceeding, this allows for a quicker dissolution and can reduce foaming. Let higher percentage gels (>1.5 %) hydrate longer than lower percentage gels.
5. Cover the mouth of the beaker with plastic wrap and make a small hole in the top to allow the solution to vent.
6. Weigh the beaker and write down the starting weight.
7. Heat the beaker in the microwave for 15-30 second intervals until the solution begins to boil.
8. Stir after each heating interval.
9. Remove the beaker from the microwave and very gently swirl.

**WARNING: THE MICROWAVED SOLUTION CAN BECOME SUPERHEATED AND FOAM OVER QUICKLY WHEN AGITATED. USE CAUTION AND ALWAYS WEAR APPROPRIATE PROTECTION.**

1. If solid agarose or gel pieces remain, return the flask to the microwave and continue heating in 15 second intervals until all product is in solution. This may take up to a few minutes depending on the strength of your microwave and the gel concentration you are making.
2. Once the gel is fully dissolved, reweigh the solution and add deionized water to the beaker to reach the starting weight. Mix thoroughly.
3. Cast the gel quickly so that the agarose does not set.
